# Recurrent paratyphoid fever A co-infected with hepatitis A reactivated chronic hepatitis B

**DOI:** 10.1186/1476-0711-13-17

**Published:** 2014-05-12

**Authors:** Yanling Liu, Yujiao Xiong, Wenxiang Huang, Bei Jia

**Affiliations:** 1 Key Laboratory of Infectious and Parasitic Diseases in Chongqing, Department of Infectious Diseases, the First Affiliated Hospital of Chongqing Medical University, No. 1 Youyi Road, Yu Zhong District Chongqing 400016, China

**Keywords:** Recurrent paratyphoid fever A, Hepatitis A, Chronic hepatitis B reactivation

## Abstract

We report here a case of recurrent paratyphoid fever A with hepatitis A co-infection in a patient with chronic hepatitis B. A 26-year-old male patient, who was a hepatitis B virus carrier, was co-infected with *Salmonella enterica* serovar Paratyphi A and hepatitis A virus. The recurrence of the paratyphoid fever may be ascribed to the coexistence of hepatitis B, a course of ceftriaxone plus levofloxacin that was too short and the insensitivity of paratyphoid fever A to levofloxacin. We find that an adequate course and dose of ceftriaxone is a better strategy for treating paratyphoid fever. Furthermore, the co-infection of paratyphoid fever with hepatitis A may stimulate cellular immunity and break immunotolerance. Thus, the administration of the anti-viral agent entecavir may greatly improve the prognosis of this patient with chronic hepatitis B, and the episodes of paratyphoid fever and hepatitis A infection prompt the use of timely antiviral therapy.

## Case presentation

More than 1 month before admission to our hospital, a 26-year-old male worker was diagnosed with paratyphoid fever due to a high fever of up to 40°C that was accompanied by dizziness, fatigue, a cough with some sputum and discomfort in the periumbilical area. *Salmonella* Paratyphi A was isolated from his blood; therefore, ceftriaxone and levofloxacin were co-administered for 7 days, and his temperature returned to normal. However, approximately 10 days after completing the treatment, he presented to a local hospital because of a recurrent high fever. The patient was given an intravenous levofloxacin infusion with little improvement in his condition, and routine blood tests revealed a white blood cell (WBC) count of 5.5 × 10^9^/L with 63% neutrophils and a platelet count of 68 × 10^9^/L. He was then transferred to our hospital. The patient had a history of hepatitis B virus beginning in childhood.

Upon admission, a physical examination revealed splenomegaly, but no bradycardia [temperature (T): 39.2°C; pulse rate (P): 99 times/min]. Laboratory studies revealed a decreased WBC count of 3.91 × 10^9^/L with 63% neutrophils and 0% eosinophils. His ALT, AST, and lactate dehydrogenase (LDH) levels were 927 U/L, 876 U/L and 224 U/L, respectively, and hepatitis B virus (HBV) DNA was present at 1.38 × 10^5^ IU/L. Blood cultures were performed twice, and a B-mode ultrasound revealed splenomegaly.

The patient received 0.3 g of levofloxacin via injection twice a day, but 2 days later, his temperature remained elevated, at approximately 39°C. Therefore, a bone marrow puncture was performed for a routine examination and culture. Viral markers for hepatitis C and E, cytomegalovirus and the Epstein-Barr virus were negative, but the marker for hepatitis A was positive (immunoglobulin M, twice; rheumatoid factor was negative). *S.* Paratyphi was isolated from his blood but not from his bone marrow, and the susceptibility test indicated that this species of bacteria had an intermediate susceptibility to ciprofloxacin. A bone marrow picture revealed no abnormalities. Therefore, a 2-g ceftriaxone injection twice a day was added to his treatment in addition to thymosin α1, which was given once every other day. Furthermore, the nucleotide analogue entecavir was given concurrently to inhibit the HBV DNA. The temperature of the patient returned to normal after 4 days of treatment with levofloxacin and ceftriaxone, and he progressively recovered his appetite and sense of well being.

Fourteen days after admission, a routine blood test revealed a leucocyte count of 6.03 × 10^9^/L (40.8% neutrophils and 4.8% eosinophils), total bilirubin and direct bilirubin counts that were within the normal range, an ALT level of 71 U/L and an AST level of 51 U/L. The blood and stool cultures were negative, and the patient was discharged in good condition. Three months later, he came back for a follow-up. The laboratory results were as follows: liver function was indicated by an ALT level of 145 U/L, anti-hepatitis A virus immunoglobulin M (anti-HAV IgM) was negative, and the HBV DNA was below the limit of detection. Six months later, his liver function totally recovered.

## Discussion

Paratyphoid fever A is a communicable disease that is transmitted through the faecal-oral route. This disease usually presents with a mild clinical manifestation that is shorter in duration and less resistant to antimicrobials than typhoid fever. The occurrence of this disease has been reported less frequently both globally and in China; however, the incidence of paratyphoid fever A has recently surpassed that of typhoid fever [1,2]. Here, we present the case of a 26-year-old man with chronic hepatitis B who was co-infected with recurrent paratyphoid fever A and hepatitis A; this case differs from previously reported cases mainly in two aspects.

As an intracellular pathogen, *Salmonella* Paratyphi is predominantly killed via the mononuclear phagocytes involved in cellular immunity. The recurrence of paratyphoid fever is closely correlated with a deficiency in cellular immunity, an inadequate dose or duration of antimicrobial treatment and a decreased susceptibility to antibiotics. Other factors, including undiagnosed, deep-seated diseases such as osteomyelitis and aortitis, are also likely to be critical for relapse [3]. The patient in this study suffered from chronic hepatitis B, and the HBV may have impaired cell-mediated immunity and favoured the relapse of *Salmonella* septicaemia. A previously reported case revealed that tuberculosis, which is caused by another intracellular pathogen, also predisposed a patient to the recurrence of *Salmonella* sepsis by interfering with the ability of macrophages to kill *Salmonella*[4]. During the first episode of paratyphoid fever for the patient in this study, both ceftriaxone and levofloxacin were used, but the course of treatment was only 7 days. These 2 drugs are the first line medications for treating adult paratyphoid fever. Although a short course of ciprofloxacin has been clinically confirmed to be efficient for curing typhoid, a longer course is likely required to cure paratyphoid because of the high rate of relapse for this disease [5,6]. When the patient’s fever returned, levofloxacin was used, which penetrates well and is usually very active against *Salmonella* species. However, the drug susceptibility test revealed that the isolate had an intermediate susceptibility to fluoroquinolones and was susceptible to ceftriaxone. *Salmonellae* that are less susceptible to fluoroquinolones have been frequently reported globally, especially in developing countries such as India and China, and this reduced susceptibility is related to a single *gyrA* mutation and an additional mutation in *parC* [ciprofloxacin minimum inhibitory concentration (MIC) ≥ 0.5 mg/L] [7-9]. Treatment failures have been described in strains that display decreased ciprofloxacin susceptibility [10]; therefore, alternative therapeutic strategies, such as arithromycin (1 g on the first day and 500 mg per day afterwards) [11] or high-dose ceftriaxone (4 g per day), are recommended for these patients. Considering the possible adverse effects of gastrointestinal disturbance and hepatic toxicity due to arithromycin, we adopted the combination of ceftriaxone (4 g per day) plus levofloxacin (0.6 g per day) for 12 days. Re-examination of the blood test results revealed normal WBC and eosinophil counts. Furthermore, the follow-up after 3 months did not indicate a relapse. Therefore, adequate cellular immunity, appropriate drug doses and a proper course of treatment are all crucial for healing paratyphoid fever. An 8-time recurrence of paratyphoid fever has been previously reported that was largely due to poor drug administration [4].

Another interesting point regarding this case is the alteration of the liver function. Three factors may have caused liver damage: *Salmonella,* the hepatitis A virus and HBV. The co-infection of paratyphoid fever with hepatitis A and/or E has been frequently reported, and in these cases, the aminotransferase levels were sharply elevated or jaundice appeared [12-14]. The incubation periods for hepatitis A and paratyphoid A are 5–50 days and 2–15 days, respectively. Therefore, these 2 diseases may occur simultaneously. It has been reported that the ratio of ALT to LDH may distinguish hepatitis caused by *Salmonella* (<4.0) and acute viral hepatitis (>5.0) [15], and the ration here was 4.13. When the patient was examined upon admission to our hospital, it is likely that his aminotransferase levels had already decreased because hepatitis A is usually a self-limiting illness. This patient, who had an intermediate increase in his HBV-DNA level, benefited because an optimal time for treating the chronic hepatitis B appeared. This patient was a young man who was in a state of immune tolerance. The co-infection of *Salmonella* and hepatitis A stimulated his cellular immune system, and the prominent elevation of the ALT and AST levels suggested that he would respond well to anti-viral therapy. The proper, early administration of anti-viral treatment to patients who have been infected with HBV since childhood will greatly decrease the possibility of developing cirrhosis and hepatocellular carcinoma (HCC). In this case, a drug with high-efficacy and a low gene-resistance barrier was more appropriate for this patient. One month after the administration of entecavir, the level of HBV DNA in the patient decreased below the detection limit. Three months later, his ALT level remained high and was even higher than when he was examined before being discharged, which suggested a good response to the entecavir treatment. At this point, his anti-HAV IgM level was already negative.

Many authors have reported that the elevation of aminotransferases in a patient with a *Salmonella* infection may signal a co-infection with other Hepadnaviridae, such as hepatitis A or E. All of these organisms are intracellular pathogens, and cellular immunity is the main defence mechanism for controlling these infections. In China, approximately 3 billion people who carry HBV, and these individuals suffer from the possibility of cellular immunity dysfunction and deficiency. Therefore, they are susceptible to infection by intracellular pathogens. The insults to the liver due to concomitant infections are usually more severe and can result in encephalopathy, liver failure or renal failure. The case presented here only had an intermediate level of liver dysfunction, which was likely due to the detection time and his age. Another aspect of this co-infection is that the concurrent infection with intracellular pathogens, such as *Salmonella* and other acute hepatitis viruses, may provide an earlier opportunity for these patients to clear the HBV with a timely anti-viral treatment due to the activation of cellular immunity, which breaks the immunotolerance barrier.

Nevertheless, the coinfection of other contagious diseases usually worsen the original disease,thus prevention rather than treatment remains the crucial way for various infectious diseases. Combination vaccines against typhoid fever and hepatitis A are already available for susceptible people [16,17].

### Consent

Written informed consent was obtained from the patient for publication of this case report and any accompanying images. A copy of the written consent is available for review by the Editor of this journal.

## Competing interests

The authors declare that they have no competing interests.

## Authors’ contributions

YL (acquisition of data, analysis and interpretation of data), YX (drafting the manuscript ), WH (has given final approval of the version to be published), BJ ( agree to be accountable for all aspects of the work in ensuring that questions related to the accuracy or integrity of any part of the work are appropriately investigated and resolved). All authors read and approved the final manuscript.
